# Ovarian hyperstimulation syndrome presenting with isolated unilateral right-side hydrothorax: A report of two cases and systematic review of the literature

**DOI:** 10.4274/tjod.galenos.2019.89656

**Published:** 2020-04-06

**Authors:** Sezcan Mümüşoğlu, Atakan Tanaçan, Volkan Turan, Gürkan Bozdağ

**Affiliations:** 1 Hacettepe Üniversitesi Tıp Fakültesi, Kadın Hastalıkları ve Doğum Anabilim Dalı, Ankara, Türkiye; 2 Üsküdar Üniversitesi Tıp Fakültesi, Kadın Hastalıkları ve Doğum Anabilim Dalı, İstanbul, Türkiye

**Keywords:** Ovarian hyperstimulation syndrome, unilateral hydrothorax, in-vitro fertilization, controlled ovarian stimulation

## Abstract

Although hydrothorax may accompany abdominal ascites in women with severe ovarian hyperstimulation syndrome (OHSS), there are few cases reported with isolated pleural effusion. Herein, we report two patients with isolated hydrothorax without any significant abdominal fluid following infertility treatment, along with a systematic review of the literature to describe risk factors for this rare entity. Two women with isolated pleural effusion without significant abdominal ascites were reported. The available literature was screened from Ovid-SP and PubMed to review OHSS cases with isolated hydrothorax. Two women aged 28 and 31 years were admitted to hospital with chest pain, tachypnea, and tachycardia after infertility treatment. They had right pleural effusion without abdominal fluid and the symptoms relieved after thoracentesis. Similar to our cases, we identified 24 case reports (n=41 women) in the literature according to eligible criteria. On the day of triggering, estradiol (E_2_) level was <4000 pg/mL in 81% of reported cases and hematocrit (HCT) was <45% in 44% of cases at the time of diagnosis. Isolated hydrothorax is an unpredictable event, which may even complicate women with low E_2_ levels or HCT concentrations. Physicians should keep in mind the possibility of isolated hydrothorax when respiratory symptoms are significant but abdominal ascites is not evident.

## Introduction

Ovarian hyperstimulation syndrome (OHSS) is an iatrogenic complication caused by ovarian stimulation procedures during infertility treatment. This syndrome occurs due to the release of cytokines from luteinized follicles, mainly vascular endothelial growth (VEGF), which induces vascular permeability, leakage of the intravascular liquid to third spaces, and a tendency to thromboembolic events^(1)^. The recognized risk factors for severe OHSS include younger age, low body mass index (BMI), high antral follicle count or anti-Müllerian hormone level, elevated peak estradiol concentration, and ≥20 oocytes collected during an in-vitro fertilization cycle^(2,3)^.

The incidence of severe OHSS is around 1% and there has been an increasing interest for novel strategies to decrease the syndrome^(1,4)^. In women with severe OHSS, although hydrothorax might already accompany significant abdominal ascites in up to 10% of cases^(2,5)^, a clinical presentation of isolated pleural effusion without any abdominal ascites is a rare entity. In that context, there is lack of data for the explanatory mechanism and risk factors that might predict the appearance of isolated hydrothorax without any significant abdominal ascites.

We performed a systematic review to describe risk factors for isolated hydrothorax and described a possible explanation that could indicate a mechanism for this clinic entity.

## Case Reports

### Case 1

A 28-year-old woman was admitted with a 3-year history of primary infertility and oligo-anovulation. Her BMI was 19.7 kg/m^2^ and her total AFC was 27. A low-dose step-up protocol with a daily dosage of 75 IU (Puregon, MSD, İstanbul, Turkey) for ovulation induction was employed. On the 9^th^ day of ovarian stimulation (OS), folliculometry with ultrasonography revealed one follicle of >17 mm, two follicles between 13-14 mm, and two follicles between 11-13 mm with an estradiol level of 2100 pg/mL. We decided to trigger (Ovitrelle, Merck, Istanbul) and perform intrauterine insemination (IUI) 36 hours later because she had only one leading follicle exceeding 17 mm.

Eight days after triggering, she was admitted to the emergency department with right chest pain, dyspnea, tachypnea, and tachycardia. The heart rate was 110/minute, blood pressure was 105/70 mm Hg, and her body temperature was 37.1°C. In a physical examination, diminished respiration sounds on the right hemi-thorax and a mild abdominal discomfort was noticed. In laboratory tests, the hemoglobin (Hb) was 15.1 g/dL, hematocrit (HCT) was 45.9%, and white blood cell (WBC) count was 16.4x10^3^. The serum electrolytes, liver enzymes, creatinine value, and cardiac panel were within the normal range; there was a slight increase in D-Dimer level up to 2.1 mg/L. Under ultrasonography, there was only minimal fluid accumulation in the recto-uterine pouch. After chest X-ray imaging (Figure 2a), computed tomography was planned, which revealed a right-sided unilateral pleural effusion with a 55-mm deep accompanying atelectasis (Figure 2b). Under fluoroscopy guidance, a total of 1500 mL serous characterized fluid was drained via transthoracic aspiration with a 22-G needle (Cook, Inc., Bloomington, IN, USA). Immediately after intervention, the symptoms were relieved and the HCT declined to 37% one day after the procedure. The patient was discharged from hospital four days later but she failed to conceive.

### Case 2

A 31-year-old woman with a normal BMI (21.4 kg/m^2^) was referred to our infertility clinic with a 4-year history of primary infertility. The AFC was 23 and the anti-müllerian hormone level was 8.49 ng/mL. Her menstrual periods were regular. A tubal patency assessment with hysterosalpingography (HSG) revealed normal findings. However, her husband had mild oligospermia. Intra-cytoplasmic sperm injection (ICSI) and embryo transfer (ET) cycle was employed (Table 1) with a daily dosage of 150 IU (Puregon, MSD, İstanbul, Turkey). On the 9^th^ day of stimulation, three follicles of ≥17 mm, eight follicles between 14-16 mm, and five follicles between 11-13 mm were identified with an estradiol level of 2890 pg/mL. We decided to trigger with a GnRH-analogue (Decapeptyl, Ferring, Kiel, Germany) with dose of 0.2 mg because she had 16 follicles ≥11 mm in a GnRH-antagonist cycle. A total of 15 oocytes were collected and 1500 IU hCG (Pregnyl, Merck, Kiel, Germany) was injected one hour after retrieval to support luteal phase^(6)^.

Two days after oocyte collection, she was admitted with severe dyspnea and tachycardia. In the laboratory tests, the Hb was 14.9 g/dL, HCT was 44.0%, and the WBC count was 15.8x10^3^. The serum electrolytes, liver enzymes, creatinine value, and cardiac panel were within the normal range. Bilateral ovaries were observed enlarged with multiple anechoic cysts in ultrasound screening, but there was no peritoneal fluid. Thorax ultrasound was performed immediately after abdominal ultrasonography after tilting the patient on her left/right side and revealed a 50-mm wide horizontal pleural effusion on the right side (Figure 2c). Chest X-ray also confirmed unilateral right-side pleural effusion and a total of 1500 mL serous characterized fluid was drained under fluoroscopy. Immediately after intervention, the symptoms were relieved. HCT declined to 39% one day after the procedure and she was discharged. ET was cancelled and all embryos were vitrified.

### Review of the Literature

Two authors (A.T. and S.M.) independently searched the PubMed & Ovid SP databases from January 1^st^, 1990, to March 1^st^, 2016. The full electronic search strategy were as described: OHS (Mesh) OR OHSS (tiab) AND isolated pleural effusion (majr) OR unilateral pleural* effusion* OR pleural effusion OR isolated hydrothorax (tiab) OR unilateral hydrothorax (tiab) OR isolated hydrothorax (tiab).

**Figure 1:**
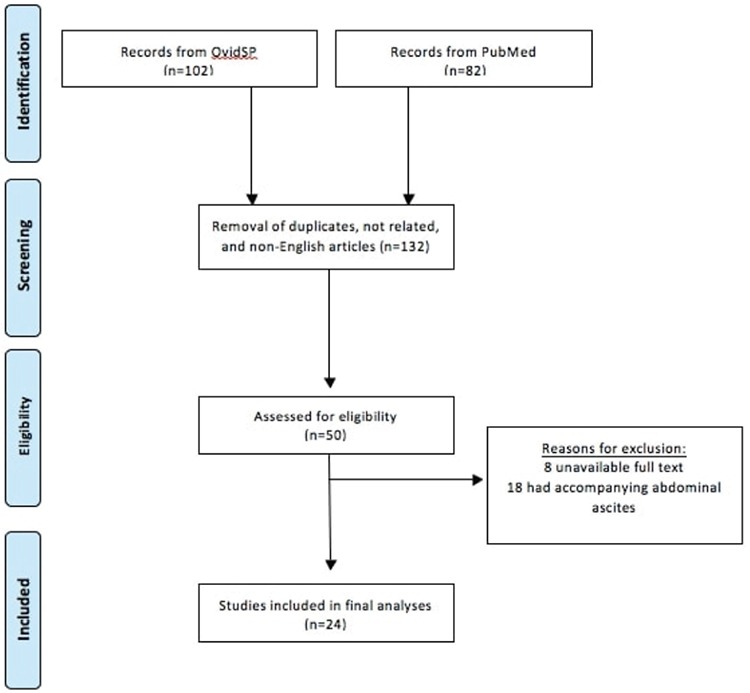
Flow diagram

**Figure 2:**
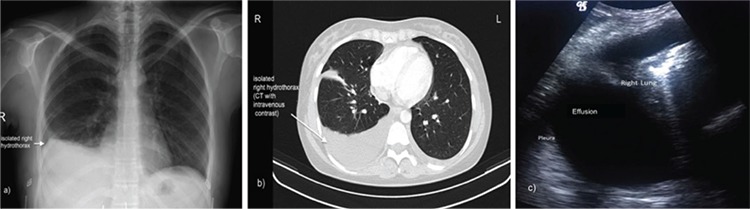
The imaging of isolated pleural effusion under X-ray (a), computed tomography (b), and (c) ultrasonography

All reported cases of isolated hydrothorax after infertility treatment were included, but accompanying ascites were taken as exclusion criteria. From the two databases, a total of 132 articles were identified. After screening from the title, abstract and text, 50 were subsequently removed due to being duplicate (n=34), not including information on OHSS (n=45), and not being written in the English language (n=3). Of the remaining 50 articles assessed for eligibility, isolated hydrothorax was not confirmed in 18 reports and data were not obtained in eight studies (Figure 1). After exclusion, the remaining 24 articles^(6,7,8,9,10,11,12,13,14,15,16,17,18,19,20,21,22,23,24,25,26,27,28,29)^ were scrutinized for the side of the isolated pleural effusion and for the patients’ information (Figure 2). Finally, two authors (S.M. and A.T.) extracted the data from full-text articles that presented information about patients’ clinical manifestation and cycle characteristic (n=24), as depicted in Table 1. Additionally, the quality assessment of the case reports was performed by using the tool suggested by Murat et al.^(30)^ in 2018. This tool was established for the evaluation of the methodologic quality of case reports and case series. Four categoric domains, which were selection, ascertainment, causality, and reporting, were assessed through eight specific questions and binary responses were scored ^(30)^. An aggregate score was preferred for all included studies in order to standardize the assessment (Supplementary file).

### Results of the literature review

A total of 41 patients with isolated hydrothorax as a single manifestation of OHSS have been reported in 24 English articles in the literature including the current report. Of them, one patient was reported twice for two consecutive IVF cycles. The median (minimum-maximum) female age and BMI of these patients were 30 (range: 21-43) years and 24 (19.5-30.4) kg/m^2^, respectively. Thirty-six of them had reported women with primary infertility, and four had been under treatment for secondary infertility. With respect to infertility etiology, polycystic ovary syndrome was the most encountered reason (n=9). Other reasons were as follows: five couples had unexplained infertility, two couples had male factor, one had hyperprolactinemia, one had endometriosis, one had tubal factor, and the remaining reports had no data (Table 2). The median (minimum-maximum) duration of infertility was 4 (minimum: 2; maximum: 10) years.

According to the available data, 14 patients were stimulated with a long agonist and five patients with an antagonist protocol in an IVF cycle. In addition, six patients had been treated with superovulation in an IUI cycle in which rescue-IVF was subsequently planned in three of them due to hyperstimulation. Seven patients were treated with ICSI and the remaining 31 with IVF. The duration of ovarian stimulation was between 7 and 13 days. The median total FSH dose consumption during ovarian stimulation was 1550 (minimum: 675, maximum: 2925) IU. All cases in the literature, except patient 2 who we presented, were prescribed recombinant or urinary hCG for triggering final oocyte maturation. The estradiol level before triggering was between 694 and 9868 pg/mL, as depicted in Table 1. In the available literature, the median number of retrieved oocytes was 19 (minimum: 8, maximum: 44), and the median number of transferred embryos was 3 (minimum: 1, maximum: 4) in the 17 cycles.

The main symptom of patients causing admittance to hospital was dyspnea accompanied by cough, orthopnea, right upper quadrant pain, and mild abdominal pain. More than half of the patients 56.1% (23/41) were examined departments other than Ob/Gyn. The presence of hydrothorax was diagnosed through chest X-ray/CT-scans, and the absence of abdominal fluid was diagnosed using ultrasonography in the cases included in this study. The minimum and maximum starting days of symptoms after ovulation triggering were 4 and 16, respectively. Available data suggest that range of Hb, HCT, and WBC count were 10.9-18.0 mg/dL, 32.0-52.0%, and 9.8x10^3^-29x10^3^, respectively. At the time of hospital admission, 92.5% (37/40) of patients had isolated right side (n=31) or right side dominant (n=6) hydrothorax. While 82.9% (34/41) of patients needed to thoracentesis either with thorax tube or needle, the remaining 17.1% (7/41) were followed conservatively. Among those patients, 14.7% (5/34) required repetitive thoracentesis. The amount of total drainage fluid once or repetitively was between 800-22,320 mL (Table 1). One patient was complicated with pulmonary emboli, and one patient needed mechanical ventilation. At the time of follow-up, all patients had full-recovery.

In terms of ET outcomes, data were available for 19 patients. Of these, two patients (10.5%) had miscarriage, and 11 patients (57%) had ongoing pregnancy or live birth.

The median quality score of the included studies was 7 (interquartile range: 1), ranging from 6 to 8. Three (12%), 11 (44%), and 11 (44%) studies had eight, seven, and six points, respectively, which indicates moderate to good quality^(30)^.

## Discussion

In both cases of isolated pleural effusion, dyspnea and tachycardia were the main symptoms without abdominal discomfort at the time of hospital admittance. Additionally, patient 2 appears to be the first case complicated by isolated pleural effusion after triggering the final oocyte maturation with a GnRH agonist, but with 1500 IU hCG for luteal rescue. The discrepancy between the severity of symptoms and visualization of only a small amount of abdominal fluid was noteworthy, suggesting that pleural effusion should be considered in such patients even when there is no apparent risk factor for OHSS. Nevertheless, at the same visit, we were able to visualize pleural effusion using ultrasonography with the same probe and hence drainage of the fluid improved the symptoms dramatically.

Of the included case series in this systematic review, most had good quality scores. Although the definitive etiopathology is not clear, it has been postulated that diaphragmatic lymphatic and multiple macroscopic defects might potentially explain the fluid leakage from the abdominal cavity to the thorax^(21)^. However, these assumptions fail to clarify cases with no significant ascites in the abdomen, but with enough pleural fluid to induce severe dyspnea and tachypnea. In that context, Kirschner et al.^(31)^ proposed that porous diaphragm syndromes might be a possible explanation for this entity. The authors constructed the hypothesis based on the presence of diaphragmatic fenestrations creating peritoneopleural communications. In this situation, the reduced hydrostatic pressure in the right upper quadrant of the abdominal cavity and intestinal sweeping would contribute to fluid accumulation in these areas. Afterwards, through the porous fenestrations located in the diaphragm, the piston-like motion of the liver capsule transfers fluid to the pleural cavity even when there is no large amount of abdominal ascites. In that context, four morphologic types of diaphragmatic pores were defined as visualized during video-thoracoscopic repair of defects in a patient with hepatic disorders-related hydrothorax^(32)^: type-1, no macroscopic defect; type-2, blebs lying on diaphragm; type-3, broken defect (fenestrations) of the diaphragm; and type-4, multiple gaps of the diaphragm. The authors reported that the amount of the fluid in the pleura was not correlated with the type of diaphragmatic defect^(32)^. Further invasive procedures were not offered to our patients presented herein because there was possibility of not achieving visualization of diaphragmatic pores, even with video-thoracoscopy.

Unilateral hydrothorax is an unexpected finding of OHSS, presenting with right side dominance. According to the available literature, pleural fluid becomes clinically apparent 5 to 16 days after oocyte retrieval and serum estradiol concentrations may be 684-9800 pg/mL, as reported with various cases (Table 1). After symptoms become significant, isolated pleural effusion might be encountered in these cases in spite of normal HCT and lack of significant abdominal ascites that might be suggestive of only mild OHSS for the physician. Therefore, physicians should consider pleural effusion in patients who mainly admit with symptoms of dyspnea within the first few weeks after triggering when there is no amount of abdominal fluid to account for the severity of symptoms. Most cases in the literature were treated with a single thoracentesis procedure. The success rate in infertility treatment, ongoing pregnancy or live birth does not appear to be negatively affected.

In the differential diagnosis of isolated hydrothorax, pulmonary embolism should be ruled out. Making a prompt diagnosis is important because the treatment and prognosis for these conditions are completely different. For physicians working in reproductive medicine, it is worth emphasizing that performing thoracic ultrasound immediately after abdominal ultrasound with the patient tilted on her sides shortens the diagnosis time. However, when ultrasonography fails to demonstrate an abdominal or pleural effusion in patients with dyspnea and tachypnea, spiral computerized tomography should be performed for a definitive diagnosis.

OHS is a life-threatening complication, as such an individualized approach including an assessment of risk factors, application of appropriate ovarian stimulation protocols, modification of regimens according to patient characteristics, and administration of an alternative to standard-dose hCG or GnRH-agonist for final oocyte maturation may decrease the risk of OHSS in patients receiving infertility treatment^(33)^.

### Study Limitations

The main limitation of the present study is not visualizing diaphragmatic pores. However, as we mentioned above, other than video-thoracoscopy, a clinically feasible non-invasive method is not available to demonstrate diaphragmatic pores.

## Conclusion

Isolated right-sided hydrothorax is a rare, unexpected, and unpredictable event, which may appear even in the setting of mild OHSS. Physicians should be aware of the possibility, diagnosis, and management of this finding. Although the hypothesis based on diaphragm pores might be a potential explanation for pathogenesis of pleural effusion, further research is required for additional risk factors such as inflammation mediators, vasoactive substances, and serosal surface defects that may accompany a porous diaphragm. Advances in imaging technology may allow physicians to demonstrate these potential pores in further studies.

## Figures and Tables

**Table 1:**
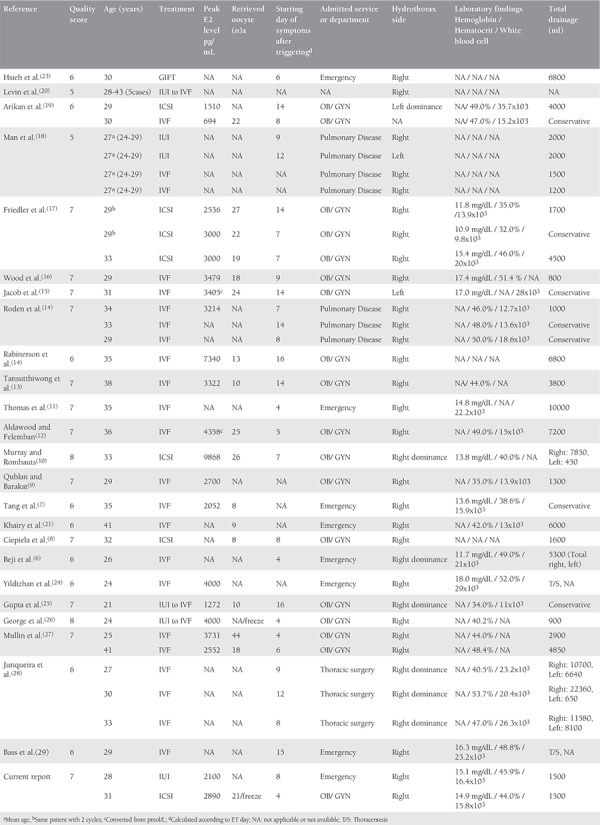
The available English literature reporting isolated hydrothorax without accompanying abdominal ascites as unusual manifestation of ovarian hyper stimulation syndrome (1990 – 2017)

**Table 2:**
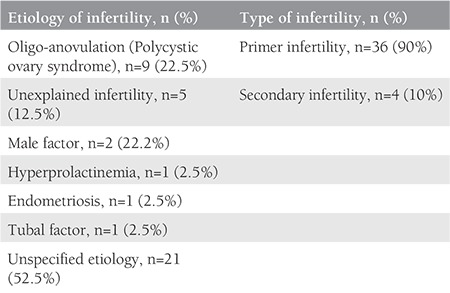
Type and etiology of the infertility of the included cases (n=41)
